# Food ileus secondary to citrus fruit associated with a Meckel’s diverticulum: a case report and review of the literature

**DOI:** 10.1093/jscr/rjy057

**Published:** 2018-03-28

**Authors:** Yukihiro Tatekawa

**Affiliations:** Department of Pediatric Surgery, Saku Central Hospital Advanced Care Center, 3400-28, Nakagomi, Saku-shi, Nagano 385-0051, Japan

## Abstract

The case of an 11-year-old boy with an orange-associated ileus in the setting of a mesodiverticular band from a Meckel’s diverticulum is reported herein. Computed tomography showed a small bowel feces sign. He underwent laparoscopic-assisted surgery, and intraoperative findings revealed a Meckel’s diverticulum associated with a mesodiverticular band distal to dilated small bowel. After resection of the Meckel’s diverticulum and inspection of the intestinal contents proximal to the obstruction, it was apparent that undigested food material was impacted proximal to the obstruction. Upon questioning postoperatively, it was revealed that the patient ate two citrus fruits with locular membranes several days prior to his clinical presentation. It was speculated that the combination of the citrus fruit impaction with the Meckel’s band led to his obstruction. He was discharged uneventfully on postoperative Day 13. It is important to avoid eating large quantities of fruit rich in fiber and also to masticate well.

## INTRODUCTION

In a review of unusual causes of small-bowel obstruction (SBO), small intestine foreign bodies are commonly reported upon. Bezoars can be found in the intestine and can cause mechanical SBO. A variety of fruit and vegetable matter has been found in phytobezoars including persimmons, orange pith, grapefruit, mango and carrots [1–10]. Prior to gastric surgery, intestinal narrowing due to congenital bands, strictures, physiologically narrowed segments and the presence of a Meckel’s diverticulum can contribute to an obstructive picture in the setting of bezoars [1–9]. In this article, we report on an ileus associated with orange consumption in a patient with a mesodiverticular band arising from a Meckel’s diverticulum.

## CASE REPORT

**Figure 1:**
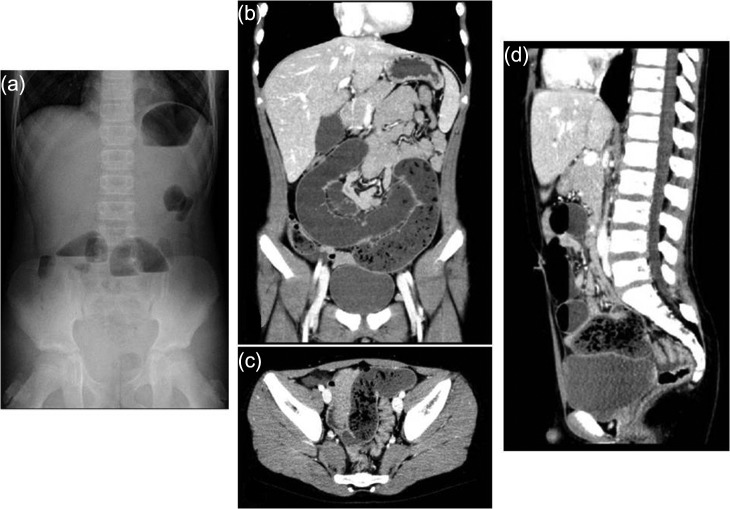
Abdominal X-ray and enhanced computed tomography. (**a**) Abdominal X-ray on admission showing multiple air-fluid levels. (**b–d**) Enhanced computed tomography showing small bowel feces sign, which is defined as the presence of feces-like material in the lumen of dilated loops of small bowel proximal to the site of obstruction.

**Figure 2:**
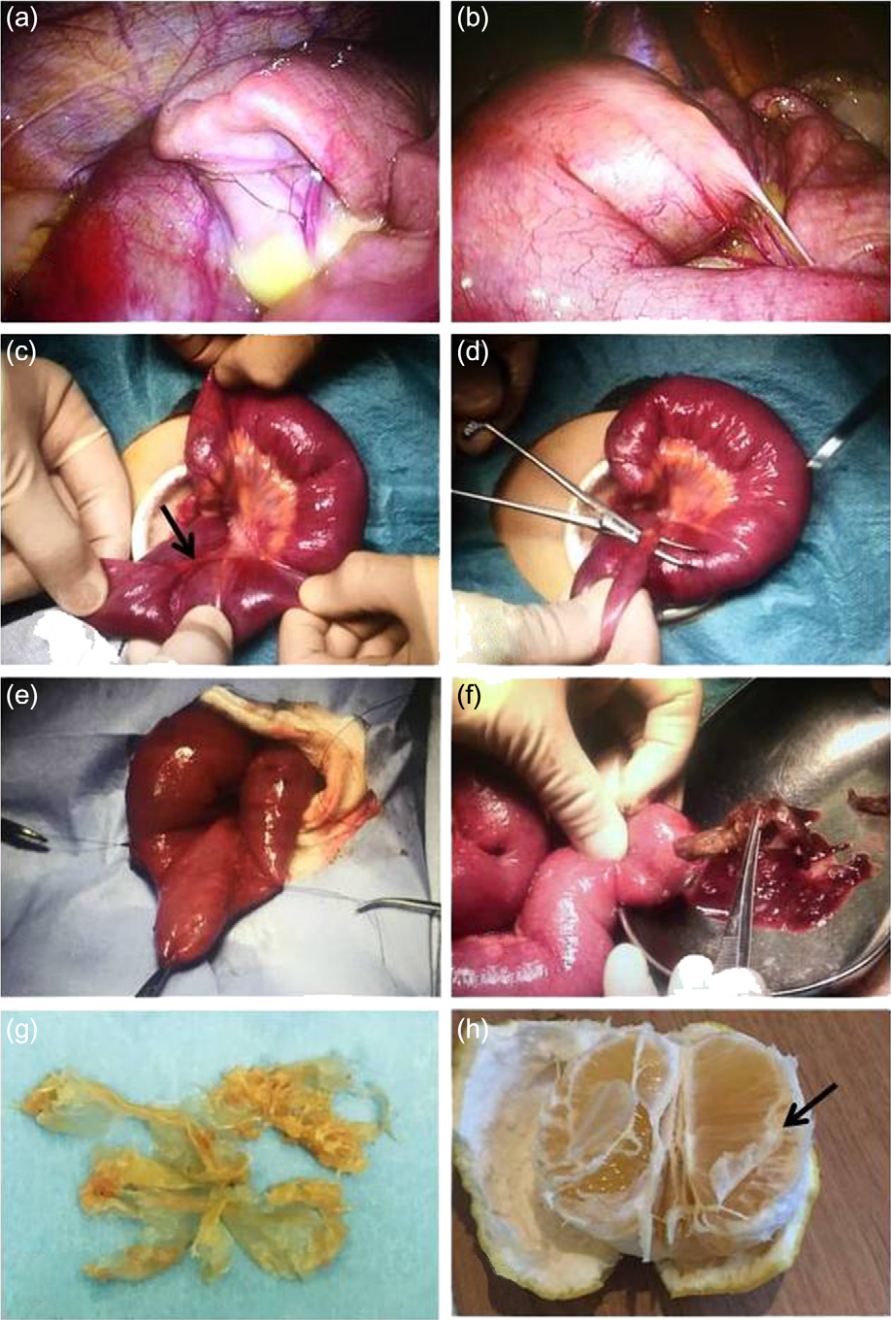
Intraoperative findings. (**a**) Intraoperative findings showing dilated and non-dilated segments of bowel in the ileum. (**b–e**) The presence of a Meckel’s diverticulum associated with a mesodiverticular band in the terminal part of dilated bowel. The Meckel’s diverticulum is tethered to the small bowel mesentery by a mesodiverticular band. (**f**) Intestinal contents from after Meckel’s diverticulum resection. (**g**) Foreign objects resembling a plastic bag, revealing the locular membranes of citrus fruit (*Citrus maxima)*. (**h**) Picture showing an example of the fruit eaten by the patient (arrow: locular membrane).

An 11-year-old boy presented with abdominal pain and emesis. Abdominal X-ray revealed multiple air-fluid levels within the small bowel (Fig. 1a). He was transferred to our hospital with the presumed diagnosis of ileus. The patient was afebrile and physical examination showed lower abdominal tenderness without peritoneal signs. Laboratory tests revealed a white blood cell (WBC) count of 13000 /μL and C-reactive protein (CRP) level of 0.07 mg/dL. Enhanced computed tomography (CT) revealed dilated and non-dilated segments of bowel showing the small bowel feces sign, which is defined by the presence of feces-like material mixed with gas bubbles in the lumen of dilated loops of small bowel proximal to the site of an obstruction (Fig. 1b–d). He underwent laparoscopic-assisted surgery. An Alexis XS wound retractor (Applied Medical Resources Corporation, Rancho Santa Margarita, CA) was inserted through a 2.0-cm vertical transumbilical incision, and a silicone cap (Free Access; TOP Corporation, Tokyo, Japan) was mounted for use as a multichannel port. Two 5-mm ports, for the flexible laparoscope and for grasping forceps, were inserted through this multichannel port. Intraoperative findings revealed dilated and non-dilated segment of bowel in the ileum (Fig. 2a). In the terminal part of dilated bowel, a Meckel’s diverticulum associated with a mesodiverticular band was noted (Fig. 2b). Upon closer inspection, the Meckel’s diverticulum was tethered to the small bowel mesentery by a mesodiverticular band and the small bowel proximal to the mesodiverticular band was dilated. We conjectured that the dilated Meckel’s diverticulum filled with intestinal contents and compressed the intestine because of the tethering to the small bowel mesentery, with resultant small bowel obstruction and dilatation of the small bowel proximal to the mesodiverticular band (Fig. 2c–e). To decompress the bowel, intestinal contents were expressed after excision of the Meckel’s diverticulum (Fig. 2f). Intestinal contents consisted of foreign objects and food debris (Fig. 2g). The Meckel’s diverticulum was resected using the Endo-GIA stapler. Upon further questioning postoperatively, it became known that the patient ate two citrus fruits (*Citrus maxima)* with locular membranes several days prior to his presentation (Fig. 2h). The foreign objects retrieved from the intestinal contents were found to be the locular membranes of said citrus fruit. He was discharged uneventfully on postoperative Day 13.

## DISCUSSION

Computed tomography is an effective tool in the evaluation of small bowel obstruction. The small bowel feces sign is an example of a finding that can be observed in small bowel obstruction on CT [10]. It is defined as the presence of feces-like material in the lumen of dilated loops of small bowel proximal to the site of obstruction. Foreign bodies in the small intestine are commonly reported on. Bezoars, for example, can cause mechanical SBO. A variety of fruit and vegetable matter has been reported in phytobezoars [1]. Phytobezoars are concretions of poorly digested fruit and vegetable fibers that are found in the alimentary tract, particularly orange pith or pulp in patients with a history of abdominal surgery and persimmon in patients without previous surgery [1–6]. In this article, the patient developed an ileus due to undigested citrus fruit. Orange-associated ileus has been most frequently described in patients after gastrectomy or gastro-jejunostomy. Delay in treatment may lead to death. The orange pith is a rich, fibrous substance based on cellulose, which is difficult to digest. It is important to avoid eating large quantities of fruit rich in fiber. It is important to masticate food well.

On the other hand, intestinal narrowing due to congenital bands, strictures, physiologically narrowed segments, and the presence of Meckel’s diverticulum can also contribute to SBO. Regarding Meckel’s diverticulum, it is the most common congenital anomaly of the gastrointestinal tract, and represents a persistent remnant of the omphalomesenteric duct. There are numerous mechanisms for bowel obstruction arising from a Meckel’s diverticulum. Obstruction can be caused by trapping of a bowel loop by a mesodiverticular band, volvulus of the diverticulum around a mesodiverticular band, and intussusception, as well as by an extension into a hernia sac (Littre’s hernia). Similarly, obstruction can be caused by trapping of a bowel loop by a mesodiverticular band [9]. In the present case, it is postulated that the dilated Meckel’s diverticulum filled with intestinal contents and compressed the intestine because of tethering to the small bowel mesentery, with a resultant small bowel obstruction.
